# Enhanced vulnerability to oxidative stress and induction of inflammatory gene expression in 3‐phosphoglycerate dehydrogenase‐deficient fibroblasts

**DOI:** 10.1002/2211-5463.12429

**Published:** 2018-05-08

**Authors:** Momoko Hamano, Yurina Haraguchi, Tomoko Sayano, Chong Zyao, Yashiho Arimoto, Yui Kawano, Kazuki Moriyasu, Miyako Udono, Yoshinori Katakura, Takuya Ogawa, Hisanori Kato, Shigeki Furuya

**Affiliations:** ^1^ Laboratory of Functional Genomics and Metabolism Department of Innovative Science and Technology for Bio‐industry Kyushu University Fukuoka Japan; ^2^ International College of Arts and Sciences Fukuoka Women's University Fukuoka Japan; ^3^ Department of Bioscience and Biotechnology Kyushu University Fukuoka Japan; ^4^ Laboratory for Molecular Membrane Neuroscience RIKEN Brain Science Institute Wako, Saitama Japan; ^5^ Department of Genetic Resources Technology Graduate School of Bioresource and Bioenvironmental Sciences Kyushu University Fukuoka Japan; ^6^ School of Pharmacy International University of Health and Welfare Tochigi Japan; ^7^ Corporate Sponsored Research Program “Food for Life”, Organization for Interdisciplinary Research Projects The University of Tokyo Japan

**Keywords:** l‐serine deficiency, oxidative stress, Phgdh, Ptgs2, Txnip

## Abstract

l‐Serine (l‐Ser) is a necessary precursor for the synthesis of proteins, lipids, glycine, cysteine, d‐serine, and tetrahydrofolate metabolites. Low l‐Ser availability activates stress responses and cell death; however, the underlying molecular mechanisms remain unclear. l‐Ser is synthesized *de novo* from 3‐phosphoglycerate with 3‐phosphoglycerate dehydrogenase (Phgdh) catalyzing the first reaction step. Here, we show that l‐Ser depletion raises intracellular H_2_O_2_ levels and enhances vulnerability to oxidative stress in Phgdh‐deficient mouse embryonic fibroblasts. These changes were associated with reduced total glutathione levels. Moreover, levels of the inflammatory markers thioredoxin‐interacting protein and prostaglandin‐endoperoxide synthase 2 were upregulated under l‐Ser‐depleted conditions; this was suppressed by the addition of *N*‐acetyl‐l‐cysteine. Thus, intracellular l‐Ser deficiency triggers an inflammatory response via increased oxidative stress, and *de novo* l‐Ser synthesis suppresses oxidative stress damage and inflammation when the external l‐Ser supply is restricted.

**Abbreviations**
Atf4: activating transcription factor 4
EMEM: Eagle's minimum essential medium
Gapdh: glyceraldehyde‐3‐phosphate dehydrogenase
GSH: glutathione
ISR: integrated stress response
MEF: mouse embryonic fibroblast
Phgdh: 3‐phosphoglycerate dehydrogenase
Ptgs2: prostaglandin‐endoperoxide synthase 2
qRT‐PCR: quantitative real‐time PCR
l‐Ser: l‐serine
Txnip: thioredoxin‐interacting protein

l‐Serine (l‐Ser) is synthesized *de novo* from 3‐phosphoglycerate via the phosphorylated pathway in which 3‐phosphoglycerate dehydrogenase (Phgdh) catalyzes the first step reaction. l‐Ser serves as a necessary precursor for the synthesis of proteins, sphingolipids, glycerophospholipids, folate metabolites, and amino acids such as glycine (Gly) and l‐cysteine (l‐Cys). Furthermore, the conversion of l‐Ser into Gly participates in the biosynthesis of purines and pyrimidines, by transferring a one‐carbon unit to tetrahydrofolate (THF). Our previous *in vivo* study demonstrated that severe l‐Ser deficiency in mice with systemic targeted disruption of *Phgdh* resulted in intrauterine growth retardation, multiple organ malformation, and embryonic lethality 1, 2. l‐Ser biosynthesis defects in humans resulting from *Phgdh* mutations were identified to be Ser synthesis disorders and Neu–Laxova syndrome, the symptoms of which are characterized by severe fetal growth retardation, microcephaly, and perinatal lethality 3, 4, 5. These findings have demonstrated that *de novo* l‐Ser synthesis is essential for embryonic development and survival in mice and humans.

We recently reported that reduced availability of intracellular l‐Ser promotes the biosynthesis and accumulation of 1‐deoxysphinganine (doxSA) and its metabolites 1‐deoxysphingolipids in mouse embryonic fibroblasts (MEFs) lacking functional *Phgdh* (KO‐MEFs) 6. The condensation of palmitoyl‐CoA with l‐Ala instead of l‐Ser generated doxSA and its biosynthesis were triggered by an increasing ratio (> 3.0) of l‐Ala to l‐Ser within the cells. doxSA elicited the activation of stress‐activated protein kinase/Jun amino‐terminal kinase and p38 mitogen‐activated protein kinase, resulting in growth arrest and death in KO‐MEFs even in the presence of l‐Ser 7. Consistent with these observations, our microarray analysis of l‐Ser‐depleted KO‐MEFs revealed that the activation of a network containing the stress‐response‐activating transcription factor ATF4–ATF3–DNA damage‐inducible transcript 3 (Ddit3) axis was most prominent among the 560 upregulated genes 8, implying that l‐Ser deficiency causes metabolic stress in KO‐MEFs. However, the causal link between reduced l‐Ser availability and vulnerability to stress remains unexplored. Here, we show that l‐Ser‐depleted KO‐MEFs are vulnerable to oxidative stress, which is accompanied by increased expression of thioredoxin‐interacting protein (Txnip), a mediator of oxidative stress to inflammation, and the proinflammatory enzyme prostaglandin‐endoperoxide synthase 2 [Ptgs2; also known as cyclooxygenase (COX) 2]. These findings suggest that l‐Ser deficiency leads to an inflammatory response through diminished protection against oxidative stress.

## Materials and methods

### Cell culture

Frozen stocks of immortalized wild‐type (WT)‐ and *Phgdh*‐knockout (KO)‐MEFs were thawed and maintained in the complete medium, high‐glucose Dulbecco's modified Eagle's medium (DMEM; Wako Pure Chemical Industries, Osaka, Japan), containing 10% FBS (Gibco, Thermo Fisher Scientific, Waltham, MA, USA) and 10 μg·mL^−1^ gentamicin (Nacalai Tesque, Kyoto, Japan) in a humidified atmosphere at 37 °C with 5% CO_2_ 2. To deprive MEFs of l‐Ser, the complete medium was replaced with Eagle's minimum essential medium (EMEM; Wako Pure Chemical Industries) supplemented with 1% FBS and 10 μg·mL^−1^ gentamicin, which contained all essential amino acids and l‐glutamine but did not include l‐Ala, l‐Asp, l‐Asn, l‐Cys, l‐Glu, Gly, l‐Pro, and l‐Ser. This medium is defined as the l‐Ser‐depleted condition, which contained 4 μm l‐Ser derived from FBS. The l‐Ser‐supplemented condition was established by adding l‐Ser (final 400 μm) to EMEM supplemented with 1% FBS and 10 μg·mL^−1^ gentamicin. In some experiments, KO‐MEF lines were retrovirally transduced with mouse *Phgdh* cDNA (KO‐MEF^*+Phgdh*^) or green fluorescent protein cDNA (Gfp; KO‐MEF^*+Gfp*^) 2 and with Atf4 short hairpin RNA (shAtf4) as previously described (T. Sayano, Y. Kawano, K. Takashima, W. Kusada, M. Udono, Y. Katakura, T. Ogawa, Y. Hirabayashi, S. Furuya, manuscript in preparation); total RNA was extracted after 6 h incubation for quantitative real‐time PCR (qRT‐PCR). To deprive MEFs of l‐Leu, the medium was replaced with DMEM (deficient in l‐Leu, l‐Arg, and l‐Lys, low glucose; Sigma‐Aldrich Japan; Tokyo, Japan) containing 1% FBS, gentamicin, 800 μm l‐Lys, and 400 μm l‐Arg, with or without 800 μm l‐Leu.

### Total glutathione quantification

Knockout‐MEFs grown in DMEM with 10% FBS and 10 μg·mL^−1^ gentamicin were replated in either l‐Ser‐supplemented (at 40% cell confluence) or l‐Ser‐depleted (at 80% cell confluence) conditions for 24 h. Cells maintained under both conditions reached 80% cell confluence and were washed with Dulbecco's phosphate‐buffered saline (DPBS) followed by scraping from the dishes with DPBS. The cell suspensions were centrifuged at 1500 ***g***, and each pellet was resuspended and lysed with 80 μL of 10 mm HCl. The lysates were alternately frozen and thawed twice, after which 20 μL of 5% (w/v) 5‐sulfosalicylic acid was added. The lysates were centrifuged at 8000 ***g*** for 10 min, and the supernatants were used for glutathione (GSH) measurement. The total GSH levels were quantified using the GSSG/GSH Quantification kit (Dojindo Laboratories, Kumamoto, Japan) according to the manufacturer's protocol, on a Multiskan™ FC microplate photometer (Thermo Fisher Scientific).

### Measurement of intracellular H_2_O_2_ generation

Knockout‐MEFs were seeded at 5 × 10^3^–1 × 10^4^ cells per well on Clear Fluorescence Black Plates (Greiner Bio‐One International GmbH, Frickenhausen, Germany) in 100 μL of complete medium and incubated overnight at 37 °C, after which the medium was replaced with EMEM containing 1% FBS with or without l‐Ser and incubated for 6 h. To detect endogenous H_2_O_2_ within cells, KO‐MEFs were washed with DPBS and incubated with 2 mm BES‐H_2_O_2_‐Ac, a cell‐permeable fluorescent probe for H_2_O_2_ (Wako Pure Chemical Industries) 9, and Hoechst 33342 (Dojindo Laboratories) for 20 min. Images were acquired using the In Cell Analyzer 1000 (GE Healthcare UK Ltd., Buckinghamshire, UK) using 360‐ and 492‐nm excitation filters, and 460‐ and 535‐nm emission filters, as previously described 10. The threshold of BES‐H_2_O_2_‐Ac intensity was set to the point at which approximately 75% of l‐Ser‐supplemented KO‐MEFs were negative, and cells were scored as positive or negative using spotfire decisionsite client 8.2 software (GE Healthcare Japan, Tokyo, Japan). This software was used to visualize and analyze the results 11, 12.

### Cell viability assay

Wild‐type‐ and KO‐MEFs were seeded at 4 × 10^4^–1 × 10^5^ cells per well in 96‐well plates in 100 μL of the complete medium and incubated overnight (12–24 h) at 37 °C. The medium was changed to EMEM containing 10% FBS and H_2_O_2_ (0.01, 0.1, 1, 5, or 10 μm), and cells were incubated for 6 h. Live cells were counted using 3‐(4,5‐dimethylthiazol‐2‐yl)‐2,5‐diphenyltetrazolium bromide (Cell Counting Kit‐7; Dojindo Laboratories), which was added to each well and incubated at 37 °C for 1 h. After gentle shaking, the absorbance of the culture medium was measured at 450 nm.

### Isolation of total RNA and qRT‐PCR

Total RNA was extracted from MEFs using an RNA Isolation Kit (Roche Diagnostics Japan, Tokyo, Japan), and 1 μg of total RNA was used for cDNA synthesis. A High Capacity cDNA Reverse Transcription Kit (Applied Biosystems, Life Technologies Japan Ltd.) was used as previously described 2, and qRT‐PCR was performed with an Applied Biosystems 7500 Real‐Time PCR System (Applied Biosystems) using THUNDERBIRD SYBR qPCR Mix (Toyobo, Osaka, Japan). The primers used were as follows: *Txnip* forward, 5′‐AGCAGGACATGGAGCAAGTT‐3′, and reverse, 5′‐TTCTTTTTCCAGCGAGGAGA‐3′; *Ptgs2* forward, 5′‐ ACAGACTGTGCCACATACTCAAGC‐3′, and reverse, 5′‐ GATACTGGAACTGCTGGTTGAAAAG‐3′; glyceraldehyde 3‐phosphate dehydrogenase (*Gapdh*) forward, 5′‐ACTCCCACTCTTCCACCTTCG‐3′, and reverse, 5′‐ATGTAGGCCATGAGGTCCACC‐3′.

### Western blot analysis

Cells were lysed to extract the total protein, which was fractionated using SDS/PAGE, and transferred to poly vinylidene difluoride membranes as previously described 2. Membranes were probed with the following primary antibodies: anti‐Txnip (1 : 100 dilution, Medical & Biological Laboratories Company, Nagoya, Japan), anti‐Cox2 (1 : 500 dilution; Cell Signaling Technology Japan K.K., Tokyo, Japan), and anti‐Gapdh (1 : 100 000 dilution; EMD Millipore, Billerica, MA, USA). Bound antibodies were visualized and quantified as previously described 2.

### Antioxidant treatments

Knockout‐MEFs were cultured in complete medium for 20 h, after which the culture medium was changed to EMEM containing 1% FBS in the absence of l‐Ser. *N*‐acetyl‐ l‐cysteine (NAC) was added to the culture medium at concentrations of 1 or 5 mm for 6 h, after which total RNA was extracted and used for qRT‐PCR as described above.

### Statistical analyses

Data were evaluated using *t*‐tests to analyze differences between two groups. To analyze differences among more than two groups, one‐way analysis of variance followed by Dunnett's *post hoc* test was used. *P*‐values < 0.05 were considered significant. Data are expressed as the means ± standard error. All statistical analyses were performed using kaleidagraph 4.0 (Synergy Software, Reading, PA, USA).

## Results

### l‐Ser deficiency reduced glutathione and increased vulnerability to oxidative stress in *Phgdh* KO‐MEFs

**Figure 1:**
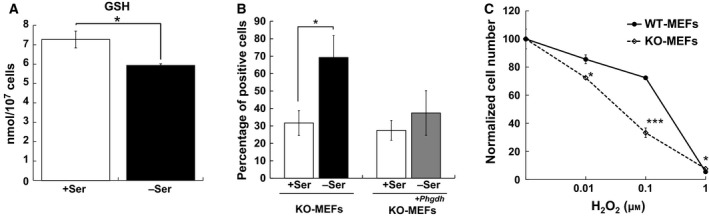
l‐Ser deficiency induces the reduction in the intracellular GSH level and resistance to oxidative stress in *Phgdh* KO‐MEFs. (A) KO‐MEFs were cultured under l‐Ser‐supplemented (+Ser) or l‐Ser‐depleted (–Ser) conditions for 24 h, and intracellular total GSH levels were measured by a GSH assay kit (*n* = 3; Student's *t*‐test, *P < 0.05). (B) KO‐MEFs and KO‐MEFs transduced with *Phgdh* (KO‐MEFs^+*Phgdh*^) were cultured under l‐Ser‐supplemented or l‐Ser‐depleted conditions for 6 h, and the production of intracellular H_2_O_2_ was analyzed using a fluorescent probe of H_2_O_2_ with In Cell Analyzer 1000 (*n* = 3; Student's *t*‐test, *P < 0.05). (C) WT‐MEFs and KO‐MEFs were cultured in complete DMEM for 16 h, and cells were cultured in EMEM containing 10% FBS supplemented with 0.01, 0.1, and 1 μm H_2_O_2_ for 6 h. Cell viability (WT‐MEFs: solid line with closed circles, KO‐MEFs: dotted line with open squares) was determined by counting the number of live cells using the MTT assay kit (WT‐MEFs, *n* = 3; KO‐MEFs, *n* = 3; Student's *t*‐test, *P < 0.05, ***P < 0.0005).

We have previously reported that l‐Ser depletion reduces the intracellular levels of Gly, Cys, and l‐Ser 2. As both Gly and Cys are necessary precursors of GSH, we compared the total GSH levels in l‐Ser‐supplemented and l‐Ser‐depleted KO‐MEFs. Figure 1A shows that intracellular GSH levels were reduced significantly in KO‐MEFs under l‐Ser‐depleted conditions compared to l‐Ser‐supplemented conditions. We then sought to determine whether endogenous production of intracellular H_2_O_2_ was altered in KO‐MEFs under l‐Ser‐depleted conditions using BES‐H_2_O_2_‐Ac, a cell‐permeable fluorescent dye for H_2_O_2_ 9. After 6‐h incubation, the percentage of BES‐H_2_O_2_ positive cells in l‐Ser‐depleted KO‐MEFs was significantly increased compared to the l‐Ser‐supplemented condition (Fig. 1B). Next, we examined the effect of H_2_O_2_ treatment on cell viability and observed that increasing the concentration of H_2_O_2_ reduced the viability of both types of MEFs (Fig. 1C). However, KO‐MEFs were more vulnerable to H_2_O_2_ than WT‐MEFs at lower H_2_O_2_ concentrations (0.1 and 0.01 μm). These observations indicated that the loss of *de novo* l‐Ser synthesis culminated in enhanced H_2_O_2_ generation and vulnerability to its oxidative stress.

### l‐Ser depletion upregulates *Txnip* and *Ptgs2* expression in KO‐MEFs

**Figure 2:**
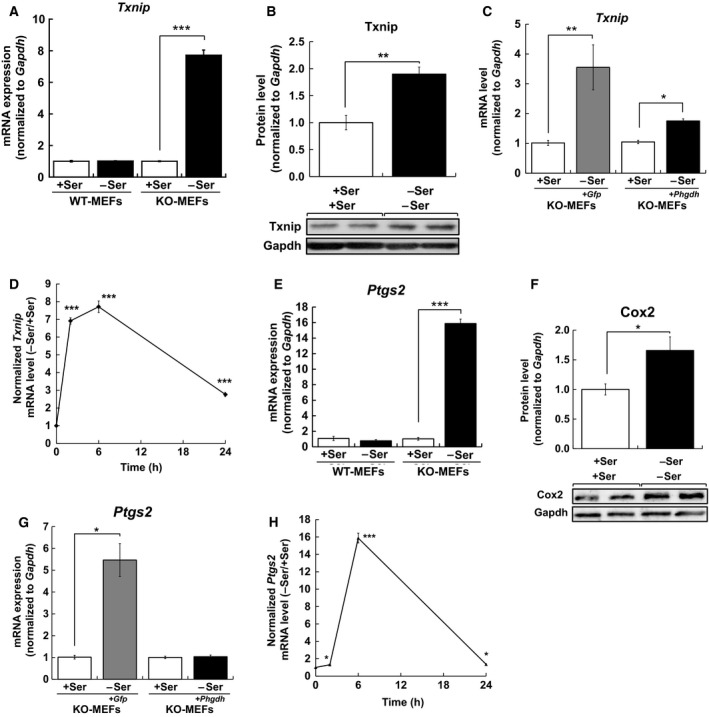
*Phgdh* deletion induced *Txnip* and *Ptgs2* expression caused by l‐Ser deficiency. (A,E) WT‐MEFs and KO‐MEFs were cultured under l‐Ser‐supplemented or l‐Ser‐depleted conditions for 6 h, and *Txnip* (A) and *Ptgs2* (E) mRNA levels were measured (WT‐MEFs, *n* = 3; KO‐MEFs, *n* = 3; Student's *t*‐test, **P < 0.005, ***P < 0.0005). (B,F) KO‐MEFs were cultured under l‐Ser‐supplemented or l‐Ser‐depleted conditions for 6 h, and Txnip (B) and Cox2 (F) protein levels were measured by western blotting and normalized to the Gapdh protein level (KO‐MEFs, *n* = 3, Student's *t*‐test, *P < 0.05, **P < 0.005). (C,H) KO‐MEFs, KO‐MEFs transduced with *Phgdh* (KO‐MEFs^+*Phgdh*^), and KO‐MEFs transduced with *Gfp* (KO‐MEFs^+*Gfp*)^ were cultured under l‐Ser‐supplemented or l‐Ser‐depleted conditions for 6 h, and *Txnip* (C) and *Ptgs2* (H) mRNA levels were measured (KO‐MEFs, *n* = 3; KO‐MEFs^+Phgdh^, *n* = 3; KO‐MEFs^+*Gfp*^, *n* = 3; Student's *t*‐test, **P < 0.005, ***P < 0.0005). (D,G) KO‐MEFs were cultured under l‐Ser‐supplemented or l‐Ser‐depleted conditions for 2 h, 6 h, and 24 h, and *Txnip* (D) and *Ptgs2* (G) mRNA levels were measured by qRT‐PCR and normalized to the *Gapdh* mRNA level (KO‐MEFs, *n* = 3, Student's *t*‐test, *P < 0.05, ***P < 0.0005).

To verify whether l‐Ser deficiency affects the oxidative stress response in KO‐MEFs, we focused on Txnip, a multifunctional protein linking oxidative stress to inflammation 12. Txnip was identified by microarray analysis as being upregulated in l‐Ser‐depleted KO‐MEFs 7, 8 and transcriptionally activated by increased H_2_O_2_ 13. First, we compared *Txnip* mRNA levels in KO‐ and WT‐MEFs under l‐Ser‐supplemented or l‐Ser‐depleted conditions. After incubation in l‐Ser‐depleted medium for 6 h, an 8‐fold increase in *Txnip* mRNA was detected in KO‐MEFs but not in WT‐MEFs (Fig. 2A). Consistently, Txnip protein expression level in l‐Ser‐depleted KO‐MEFs significantly increased to 1.8‐fold higher than that in l‐Ser‐supplemented KO‐MEFs (Fig. 2B). To examine whether *Txnip* mRNA induction was due to *Phgdh* deletion, we measured the *Txnip* mRNA levels in KO‐MEFs^*+Phgdh*^ and in KO‐MEFs^*+Gfp*^ under l‐Ser‐depleted conditions. Viral transduction of *Phgdh*, but not *Gfp*, suppressed *Txnip* mRNA induction under l‐Ser‐depleted conditions (Fig. 2C), indicating that loss of *Phgdh* was primarily responsible for *Txnip* induction under l‐Ser‐depleted conditions. Time course analysis of *Txnip* mRNA expression demonstrated a sharp 7‐fold increase as early as 2 h after the exposure of l‐Ser‐depleted medium to KO‐MEFs, and a significant 3‐fold increase 24 h after exposure (Fig. 2D).

Next, we tested whether the expression of *Ptgs2*, a proinflammatory enzyme, was also induced in l‐Ser‐depleted KO‐MEFs, because *Txnip* participates in the regulation of *Ptgs2* expression 14, 15. We compared *Ptgs2* mRNA levels in KO‐MEFs and found that after 6‐h incubation, a substantial 16‐fold increase in *Ptgs2* mRNA was detected in the l‐Ser‐depleted condition compared to the l‐Ser‐supplemented condition, while WT‐MEFs did not show such an increase in l‐Ser‐depleted conditions (Fig. 2E). Accordingly, a significant increase in Ptgs2 protein was observed in l‐Ser‐depleted KO‐MEFs compared to those in l‐Ser‐supplemented conditions (Fig. 2F). As with *Txnip* (Fig. 2C), viral transduction of *Phgdh* cDNA but not *Gfp* suppressed *Ptgs2* mRNA induction (Fig. 2G). Time course analysis of *Ptgs2* mRNA expression demonstrated a subtle but significant 1.3‐fold increase after 2‐h incubation of KO‐MEFs in l‐Ser‐depleted medium, which reached a plateau after 6‐h incubation, and retained a 1.4‐fold increase even after 24 h (Fig. 2H). These observations indicated that reduced l‐Ser availability caused by *Phgdh* disruption results in the upregulation of both mRNA and protein levels of *Txnip* and *Ptgs2* within 6 h.

### Transcriptional activation of *Txnip* and *Ptgs2* is independent of the integrated stress response pathway

**Figure 3:**
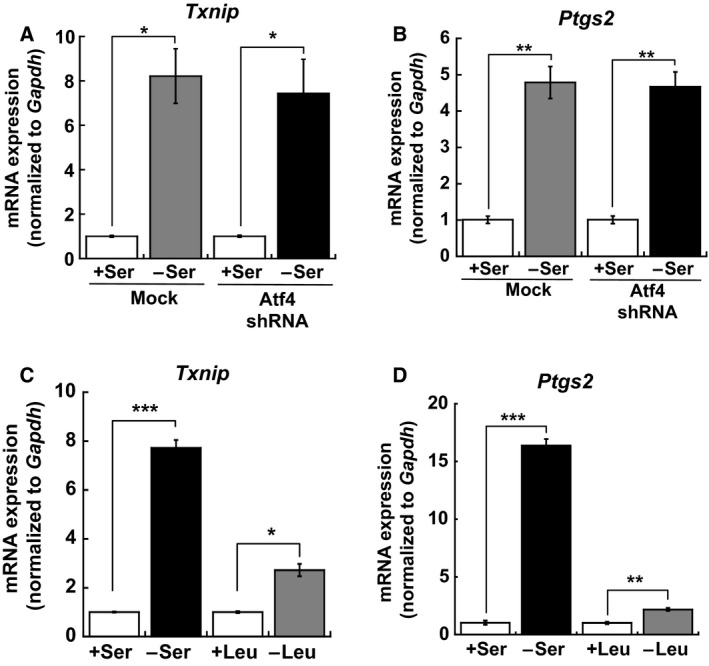
*Txnip* and *Ptgs2* induction is not associated with the ISR pathway activated by amino acid deficiency in l‐Ser‐depleted KO‐MEFs. (A,B) Mock‐ and shAtf4‐transduced KO‐MEFs were cultured under l‐Ser‐supplemented or l‐Ser‐depleted conditions for 6 h, and *Txnip* (A) and *Ptgs2* (B) mRNA levels were measured (mock‐transduced KO‐MEFs, *n* = 3; shAtf4‐transduced KO‐MEFs, *n* = 3; Student's *t*‐test, *P < 0.05, **P < 0.005). (C,D) KO‐MEFs were cultured under l‐Leu‐supplemented or l‐Leu‐depleted conditions for 6 h, and *Txnip* (C) and *Ptgs2* (D) mRNA levels were measured (KO‐MEFs, *n* = 3, Student's *t*‐test, *P < 0.05, **P < 0.005, ***P < 0.0005).

To gain insight into the underlying molecular mechanisms by which *Txnip* expression was upregulated under l‐Ser‐depleted conditions, we examined whether the integrated stress response (ISR) pathway, which is activated by amino acid deficiency, regulated *Txnip* expression in l‐Ser‐depleted KO‐MEFs. It has been well documented that deprivation of one or more amino acids can induce the activation of the ISR pathway, which results in enhanced phosphorylation of the translation initiation factor 2α and subsequent increased expression of the transcription factor Atf4 16, 17. As a result of amino acid deprivation, Atf4 target genes are substantially induced 18. As we have demonstrated that l‐Ser depletion in KO‐MEFs causes a robust increase in Atf4 protein expression (Sayano *et al*., manuscript in preparation) and upregulation of several Atf4‐target genes 2, 7, we sought to determine whether *Txnip* and *Ptgs2* induction was regulated by *Atf4* via the ISR pathway. To evaluate the functional involvement of *Atf4* in KO‐MEF transcription, we generated shRNA‐mediated *Atf4* knockdown (KD)‐KO‐MEFs, in which Atf4 protein expression was suppressed by 0.4‐fold under l‐Ser‐depleted conditions compared to mock‐treated KO‐MEFs (Sayano *et al*., manuscript in preparation). As shown in Fig. 3A,B, *Txnip* and *Ptgs2* induction in *Atf4* KD‐KO‐MEFs was unchanged compared to mock KO‐MEFs under l‐Ser‐depleted conditions, suggesting that *Atf4* and the ISR pathway did not play a role in *Txnip* and *Ptgs2* induction in l‐Ser‐depleted KO‐MEFs. The ISR pathway is activated in response to the deficiency of indispensable amino acids, especially l‐Leu 16, 17, 18.

We then investigated whether the depletion of the indispensable amino acid l‐Leu could induce *Txnip* and *Ptgs2* expression in KO‐MEFs. Figure 3C,D shows that l‐Leu depletion elicited significant increases in *Txnip* and *Ptgs2* expression, although the magnitudes of induction were lower than those observed during l‐Ser‐depleted conditions (Fig. 3C,D). These observations suggest that l‐Ser deficiency plays a more profound role in *Txnip* and *Ptgs2* expression than l‐Leu deficiency.

### Antioxidant addition suppresses *Txnip* and *Ptgs2* expression caused by l‐Ser depletion

**Figure 4:**
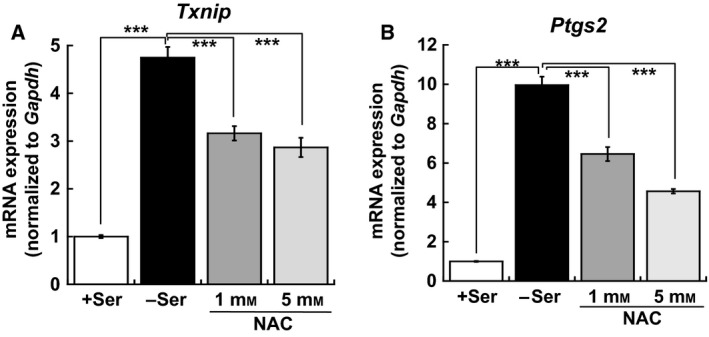
Antioxidant NAC treatment suppresses *Txnip* and *Ptgs2* induction caused by l‐Ser deficiency. (A,B) KO‐MEFs were cultured under l‐Ser‐supplemented or l‐Ser‐depleted conditions in the presence of 1 mm or 5 mm NAC for 6 h, and *Txnip* (A) and *Ptgs2* (B) mRNA levels were measured (KO‐MEFs, *n* = 3; Dunnett's *post hoc* test, ***P < 0.0005).

To clarify the upstream mechanism underlying the upregulation of *Txnip* and *Ptgs2* seen in l‐Ser‐deficient KO‐MEFs, we examined the effects of antioxidant treatment on mRNA expression. The addition of NAC, a GSH precursor, caused significant suppression of mRNA expression of both *Txnip* and *Ptgs2* in KO‐MEFs under l‐Ser‐depleted conditions (Fig. 4A,B). These results suggest that increasing oxidative stress elicited by l‐Ser depletion causes the upregulation of *Txnip* and *Ptgs2* in KO‐MEFs.

## Discussion

This study demonstrated that intracellular l‐Ser deficiency caused by *Phgdh* deletion and external l‐Ser depletion elicited increased vulnerability to oxidative stress via the reduction in GSH, which led to the induction of *Txnip* and *Ptgs2* expression in nonmalignant MEFs. GSH is synthesized from l‐Glu, l‐Cys, and Gly and prevents damage to cellular components against oxidative stress generated by intracellular reactive oxygen species (ROS). Several studies in cancer cells have reported that the intracellular levels of l‐Ser, Gly, and GSH decrease after exposure to H_2_O_2_ 19, and l‐Ser and Gly depletion leads to decreased GSH levels and increased cell death in p53^−/−^ or p21^−/−^ HCT116 cells 20. Our observations in KO‐MEFs, indicating that the *Phgdh*‐dependent l‐Ser biosynthetic pathway plays a primary role for maintaining the intracellular GSH level and preventing cell death under nutritional stress conditions, are consistent with these reports 21, 22.

We demonstrated that l‐Ser depletion induced *Txnip* expression in KO‐MEFs. *Txnip*, also known as vitamin D3‐upregulated protein 1, was originally identified as a negative regulator of thioredoxin 1/2 (Trx), a key sensor of cellular redox status that regulates protection against oxidative stress. The Trx–Txnip complex is a critical regulator of intra‐ and extracellular redox signaling and ROS 23. *Txnip* expression is upregulated by ROS 12, 24, 25 and oxidative stress caused by ischemic–reperfusive injuries 26, 27. This study showed that *Txnip* expression was induced by l‐Ser depletion in KO‐MEFs, and this was inhibited by viral transduction of *Phgdh* cDNA (Fig. 2A–C). It is well documented that *Txnip* links oxidative stress to inflammation by activating NLR family pyrin domain containing 3 11, 28, 29, and participates in the upregulation of *Ptgs2* expression 13, 14. The ISR pathway is an oxidative stress defense mechanism in response to essential amino acid deficiency 16. This study showed that *Txnip* induction caused by l‐Ser deficiency was not suppressed by *Atf4* KD in l‐Ser‐depleted KO‐MEFs (Fig. 3A), indicating that *Txnip* induction is independent of the ISR pathway. *Txnip* induction by l‐Ser depletion was significantly suppressed by the addition of an antioxidant (Fig. 4A). These observations suggest that *Txnip* expression is increased by reduced l‐Ser availability, linking aberrant redox regulation and the induction of an inflammatory response to l‐Ser deficiency that is independent of the ISR pathway.

*Txnip* affects the inflammatory response and cell death signaling by regulating the cellular redox status 30, and loss of *Txnip* can lead to the proliferation of cancer cells 31. We previously reported that l‐Ser‐depleted KO‐MEFs exhibited cell growth arrest and increased cell death after 96‐h incubation, which was associated with diminished mRNA translation and aberrant sphingolipid metabolism 2, 6, 7. In addition to GSH, proteins, and lipids, *Phgdh* expression has been proved to be critical to maintain molecules important for cell proliferation, including reduced nicotinamide adenine dinucleotide phosphate 32, purine nucleotides 33, and THF metabolites 18, 33. Taken together, the present study implies that the loss of *de novo* l‐Ser biosynthesis leads to cell proliferation arrest, followed by oxidative stress and inflammation, which seems to be a more severe cellular consequence compared to the loss of other nonessential amino acids but comparable to the severe phenotypes caused by the genetic deficiency of Gln 34. We demonstrated that L‐Ser deficiency promotes the biosynthesis and accumulation of doxSA, which can activate p38 MAPK in L‐Ser‐depleted KO‐MEFs 7. As Txnip expression was induced by H_2_O_2_ via p38 MAPK activation in human aortic smooth muscle cells 35, further study is needed to clarify whether p38 MAPK regulates Txnip induction in l‐Ser‐depleted KO‐MEFs. These insights might contribute to the elucidation of the pathobiology of patients with Neu–Laxova syndrome/l‐Ser deficiency disorders 3, 4, 5, 34 or diseases associated with increased *Txnip* expression such as diabetes and obesity 23.

## Author contributions

MH and SF designed the study and wrote the manuscript. MH, YH, TS, YA, CZ, YK, and KM performed the experiments. TS, MU, and YK prepared contributed *Atf4*‐transduced KO‐MEFs. TO prepared contributed *Phgdh*‐transduced KO‐MEFs. HK supervised contributed to the analysis of microarray data.
